# The ultrastructure of a stria vascularis in the auditory organ of the cuban crocodile (*Crocodylus rhombifer*)

**DOI:** 10.3389/fcell.2023.1129074

**Published:** 2023-02-20

**Authors:** Hao Li, Karin Staxäng, Monika Hodik, Karl-Gunnar Melkersson, Helge Rask-Andersen

**Affiliations:** ^1^ Department of Surgical Sciences, Head and Neck Surgery, Section of Otolaryngology, Uppsala University Hospital, Uppsala, Sweden; ^2^ The Rudbeck TEM laboratory, Uppsala University, Uppsala, Sweden; ^3^ Curator of reptiles, Kolmårdens Tropicarium, Kolmården, Sweden

**Keywords:** crocodilian, electron microscopy, auditory organ, stria vascularis, gap junctions crocodiles and stria vascularis

## Abstract

**Background:** An endocochlear potential (EP) exists in the mammalian cochlea generated by the stria vascularis and an associated fibrocyte network. It plays an essential role for sensory cell function and hearing sensitivity. In non-mammalian ectothermic animals the endocochlear potential is low and its origin somewhat unclear. In this study, we explored the crocodilian auditory organ and describe the fine structure of a stria vascularis epithelium that has not been verified in birds.

**Material and Methods:** Three Cuban crocodiles (*Crocodylus rhombifer*) were analyzed with light and transmission electron microscopy. The ears were fixed in glutaraldehyde The temporal bones were drilled out and decalcified. The ears were dehydrated, and embedded and was followed by semi-thin and thin sectioning.

**Results:** The fine structure of the crocodile auditory organ including the papilla basilaris and endolymph system was outlined. The upper roof of the endolymph compartment was specialized into a Reissner membrane and tegmentum vasculosum. At the lateral limbus an organized, multilayered, vascularized epithelium or stria vascularis was identified.

**Discussion:** Electron microscopy demonstrates that the auditory organ in *Crocodylus rhombifer*, unlike in birds, contains a stria vascularis epithelium separate from the tegmentum vasculosum. It is believed to secrete endolymph and to generate a low grade endocochlear potential. It may regulate endolymph composition and optimize hearing sensitivity alongside the tegmentum vasculosum. It could represent a parallel evolution essential for the adaptation of crocodiles to their diverse habitats.

## Introduction

Hearing in amniotes depends on the activation of a set of inner ear hair cells submersed in a fluid called endolymph that is rich in potassium ions. In the mammalian cochlea there is a high positive endo-cochlear potential (EP), that is essential for the high sensitivity of the auditory receptors to transduce mechanical energy into electrical signals, especially at high frequencies (Russell, 1983; Russell et al., 1986; Hibino and Kurachi, 2006; Hibino et al., 2010). In the mammalian auditory organ, the EP is thought to be generated by the stria vascularis (SV); a vascularized multicellular epithelium associated with a fibrocyte network in the lateral wall of the cochlea (Békésy and Sy, 1952; Tasaki and Spyropoulos, 1959; Salt et al., 1987; Spicer and Schulte, 1996). The cells are endowed with a plethora of ion channels and transporters. Schmidt (1963) characterized three types of EPs in ectothermic, avian, and mammalian auditory organs. Frogs, turtles, snakes, lizards, and crocodilians generally have a low EP, around +2 to +7 mV (Schmidt and Fernandez, 1962), while birds have a somewhat higher EP between +10 and +20 mV (Schmidt, 1963; Sauer et al., 1999) and therian mammals +80 to +90 mV (Wangemann, 2002; Wilms et al., 2016). A SV is well developed in marsupials that have a cochlea similar to eutherian mammals (Aitkin et al., 1979) and is remarkably developed in monotremes, such as the duckbill and spiny anteaters (Tachyglossidae). The monotremes seem to have both reptilian features such as egg-laying and some mammalian organization of the organ of Corti. They hold a lagena at the distal end of the cochlear duct and have a well-developed SV with associated capillary network (Pritchard, 1881; Smith and Takasaka, 1971; Fritzsch et al., 2013; Schultz et al., 2017). In non-mammalian tetrapods including birds, the Reissner membrane contains a folded single-layer epithelium named tegmentum vasculosum (TV) overlying the basilar papilla between the scala media and vestibule cells (Deiters, 1860; Retzius, 1884; Satoh, 1917; Kolmer, 1928; Jahnke et al., 1969; Dohlman, 1970; Rosenhall, 1971; Smith and Takasaka, 1971; Tanaka and Smith, 1978; Wilms et al., 2016; Pfaff et al., 2019). If it is homologous with the mammalian SV responsible for the production of EP and secretion of endolymph is not known.

Recently, we analyzed the auditory organ in two species of the crocodilian family (*Crocodilys rhombifer and Osteolaemus tetraspis*), using transmission electron microscopy (TEM) and immunohistochemistry (Li et al., 2022). Besides a highly specialized TV, we also detected a separate multicellular epithelium located at the abneural side of the papilla basilaris with intra-epithelial capillaries. It is reminiscent of a mammalian SV and we speculate that, unlike many other non-mammalian species, the crocodilians also possess a functionally equivalent SV. It could represent an early evolutionary transformation of structure resulting in an analogous organization of endolymph secreting epithelia and generating EP. A survey of literature showed that an epithelium reminding of a SV tissue was already described in crocodilians by Gustaf Retzius (1884) at the lateral limbus in the *Alligator mississippiensis* (Retzius, 1884) and also by Ganeshina (1990) in the *Caiman crocodylus* (Ganeshina, 1990). No similar epithelium was described in birds to our knowledge. Hasse (1873) studied the inner ear in two crocodilian specimens (*Crocodylus niloticus*) but there is no mention of a SV (Hasse, 1873). Anatomical analyses, including electron microscopy, of the crocodilian auditory organ have been performed by several authors without description of SV (Baird, 1974; Boord, 1961; Baird, 1974; Leake, 1977; Drenckhahn et al., 1991; Gleich and Manley, 2000; von Düring et al., 1974; Li et al., 2022). The aim of this presentation was to give a detailed portrayal of the fine structure of the SV in the Cuban crocodile (Crocodylus rhombifer). Electron microscopic imaging of the crocodilian SV has not been presented before to our knowledge. Hopefully it could broaden our understanding of the ancient progression and evolution of this important part of the inner ear across different vertebrates.

## Material and methods

### Transmission electron microscopy (TEM)

Two male specimens of the Cuban crocodile (*Crocodylus rhombifer*) weighing 250 gm were anesthetized using Ketamine 5 mg and Medetomidine 0.05 mg and euthanized using an intracardial injection of T-61 0.4 mL (Merck Animal Health. 200 mg embutramide for narcotic action and 50 mg mebezonium iodide for curariform action and 5 mg tetracaine hydrochloride, in aqueous solution). The skull was separated, and the temporal bones were removed using an oscillating saw. The eardrum and the columella were removed and the ears immersed in 2.5% glutaraldehyde and 1% PFA in 2.5% phosphate buffer. The temporal bones were placed in fixative for 48 h and in 0.1 M Na-EDTA for 3 weeks. Thereafter, the surrounding bone was further removed and the ears placed in 1% osmium tetroxide. The specimens were dehydrated in graded ethanol and embedded in Epon. The embedded specimens were divided into different pieces and mounted for semi-sectioning (1 µm thick). Sections were stained in toluidine blue and photographed. Areas of interest were thin-sectioned, and the sections were stained in lead citrate and uranyl acetate and examined at 80 kV in a Tecnai G2 Spirit TEM (Thermo Fisher/FEI Company, Eindhoven, Netherlands). Images were acquired with an ORIUS™ SC200 CCD camera (Gatan Inc. Pleasanton, CA, United States), using the Gatan Digital Micrograph software. A human SV from a cochlea taken out for an earlier study was used for analysis and comparison (Rask-Andersen et al., 2000).

## Results

### Light microscopy

**FIGURE 1:**
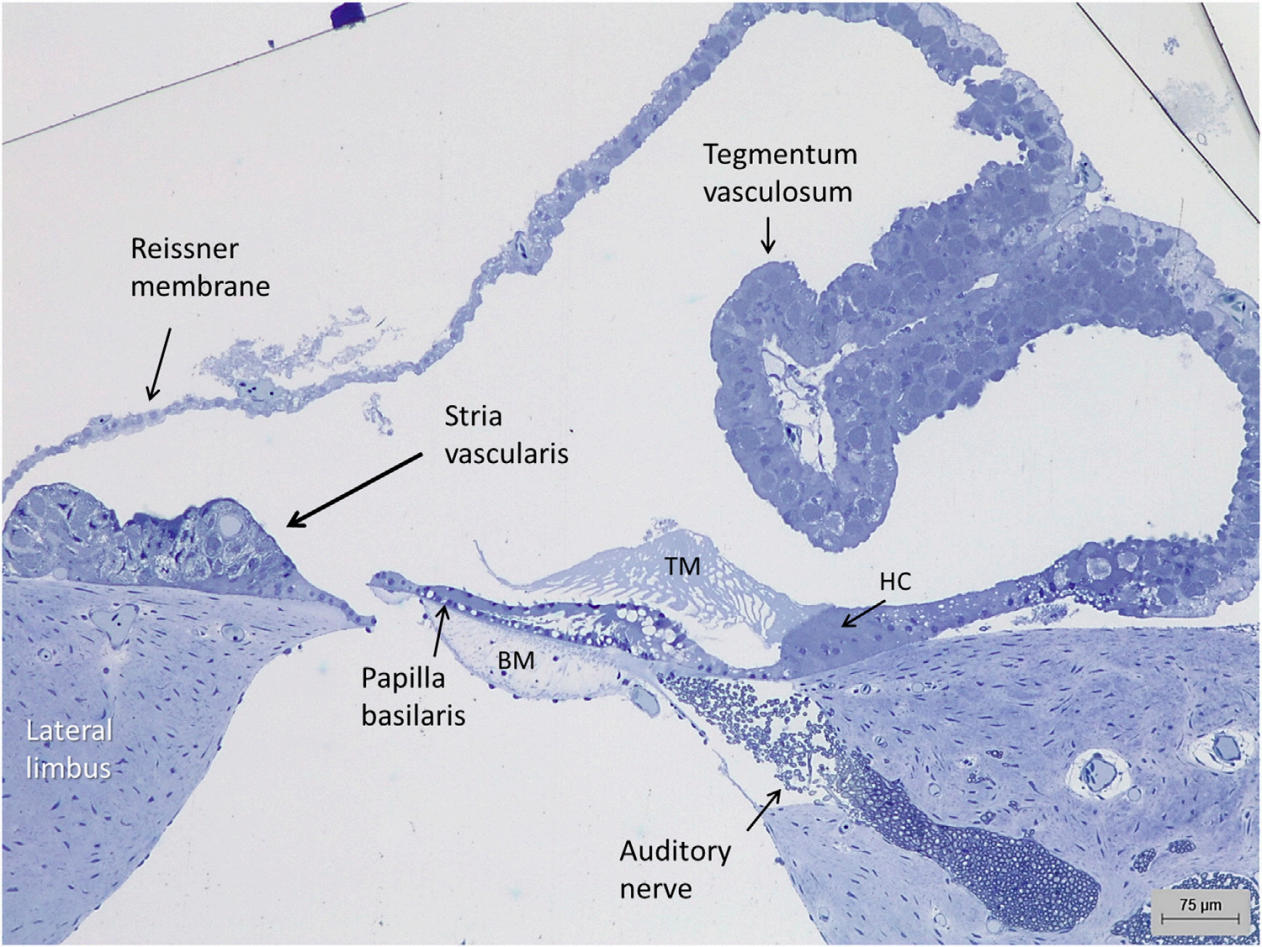
Light microscopy of the auditory organ in the *Crocodylus rhombifer*. A stria vascularis is located at the lateral limbus. A tegmentum vasculosum epithelium evaginates into the endolymphatic space. HC, homogene cells; TM, tectorial membrane; BM, basilar membrane.

Semi-thin cross sections of the crocodilian auditory organ showed the scala media with papilla basilaris, tectorial membrane, and the epithelium overlying the organ separating endolymph from the scala vestibule perilymph (Figure 1). A Reissner membrane varied in morphology from a thin or cubic single layer to a folded, epithelium with many subepithelial blood vessels representing a tegmentum vasculosum (TV). Reissner membrane was composed of dark and light cells reaching the surface.

**FIGURE 2:**
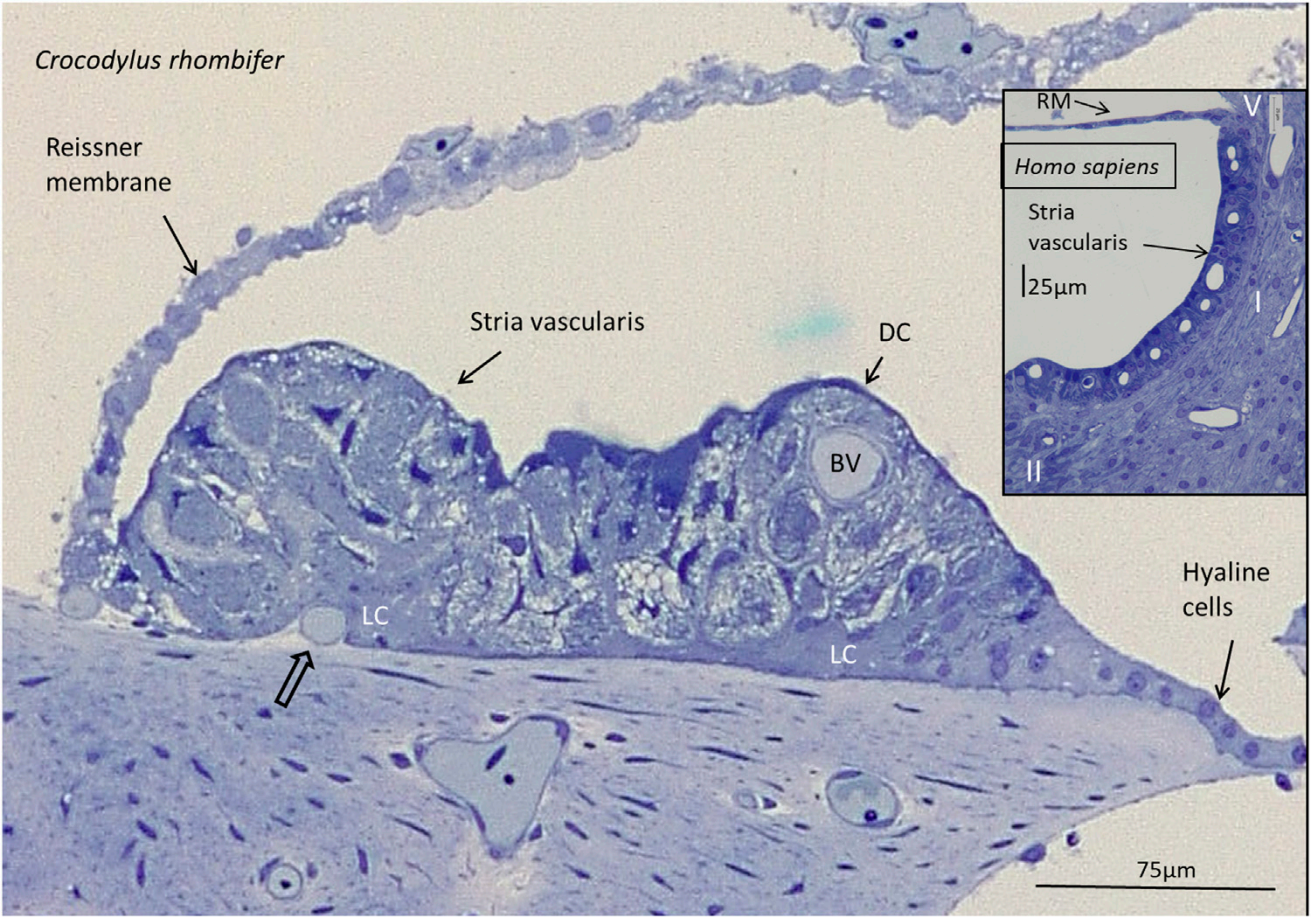
Light microscopy of the stria vascularis in the *Crocodylus rhombifer* at the lateral fibrocartilaginous plate. There is a multi-layered epithelium with apical dark cells (DC) and basally light cells (LC). The epithelium contains blood vessels (BV). A vessel is seen to merge with the epithelium (open arrow). Inset shows the SV in the basal turn of a human cochlea for comparison. It contains marginal, intermediate and basal cells. Together with the lateral fibrocytes, they are believed to secrete and recirculate K^+^ ions and generate an endocochlear (EP) essential for sensory hair cell transduction. Type I, II, and V fibrocytes are shown of the five different types (I–V). RM, Reissner membrane.

Lateral to the hyaline cells, there was an epithelial ridge extending approximately 300 µm to the Reissner membrane (Figure 1). It was multi-layered and contained dark cells facing endolymph that had both a densely stained cytoplasm and cell nucleus. They were both flat and columnar and had an irregular basal and basolateral cell coat. Basal light cells had a less densely stained cytoplasm and cell nucleus. The light cells ramified and extended from the limbus up against the dark cells. Several darkly stained, branched cells were located deep in the epithelium. In contrast to the TV, this epithelium contained blood vessels and thereby reminded one of a mammalian SV. These vessels entered through the basal lamina and branched from a richly vascularized lateral limbus (Figure 2). The SV contained remarkable spherical intercellular spaces having a diameter around 20 µm. They appeared close to the epithelial surface but also deeper in the epithelium near the basal lamina. The spaces seemed to be shaped by the light cells and contained densely stained dark cells with foliated cell processes.

### TEM

**FIGURE 3:**
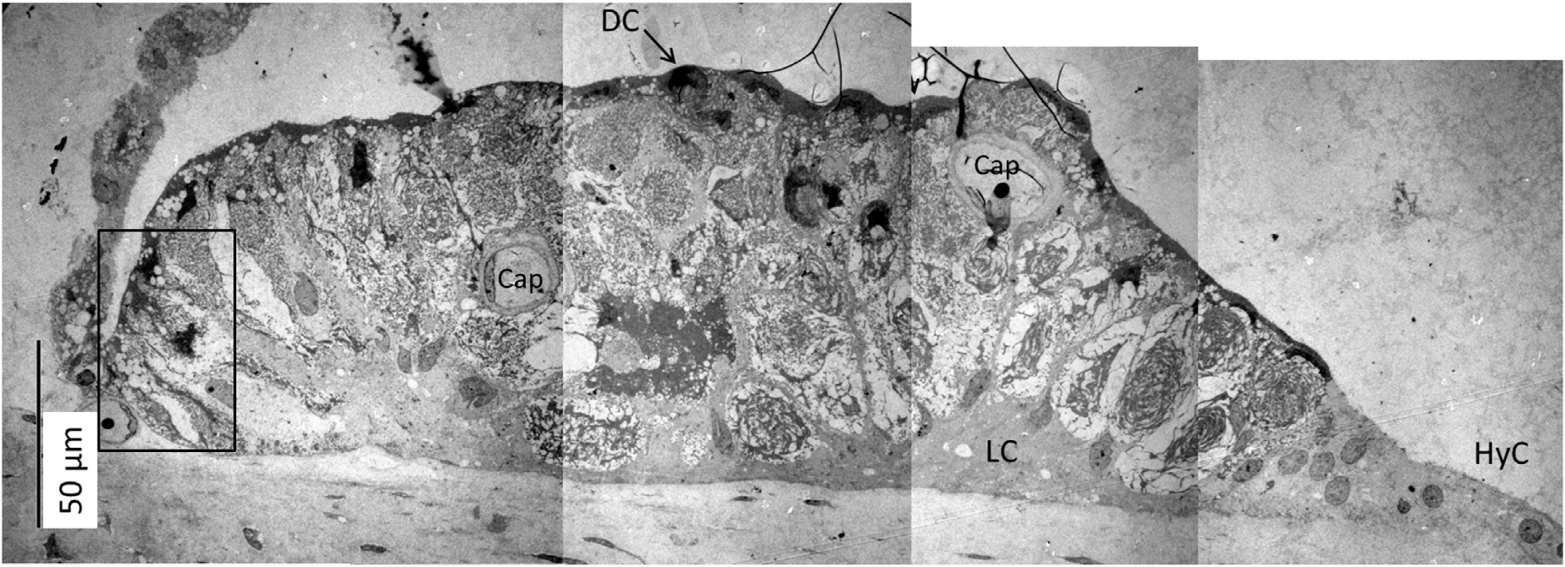
Electron micrograph of a crocodilian stria vascularis showing apical dark (DC) and basally light cells (LC). The epithelium contains capillaries (Cap). The LCs form a network with intercellular spaces containing DC enfoldings. The DCs contain clear secretory-like vesicles near the endolymph lumen. Framed area shows a DC extending basally and is magnified in Figure 13. HyC, hyaline cells; RM, Reissner membrane.

**FIGURE 4:**
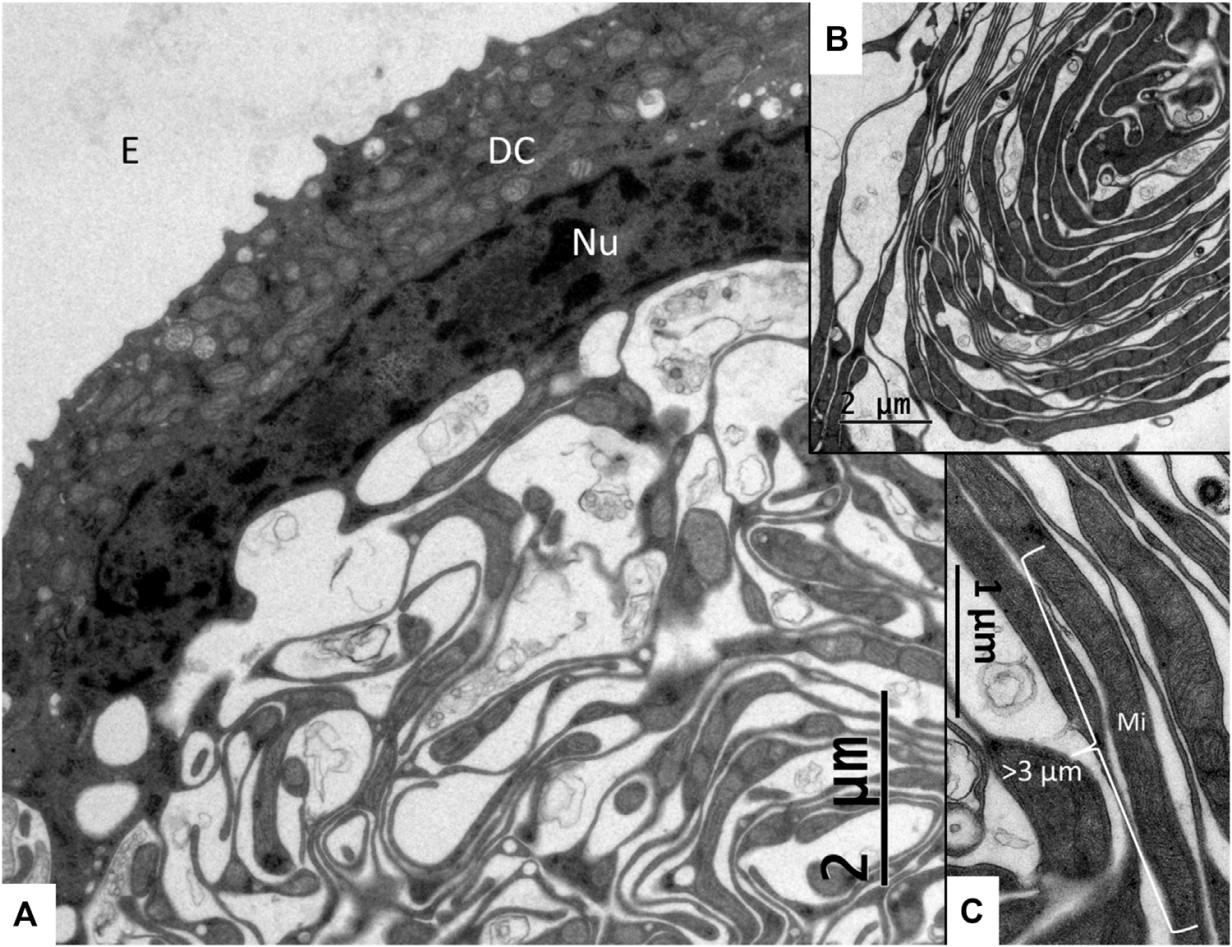
**(A)** A dark cell (DC) contains a rich number of mitochondria with basal, mitochondria-rich enfoldings. **(B)** A coil of dark cell lamellae protruding into the intercellular space. **(C)** Higher magnification of dark cell enfoldings showing lamella with long mitochondria (Mi). Nu, Nucleus; E, endolymph.

**FIGURE 5:**
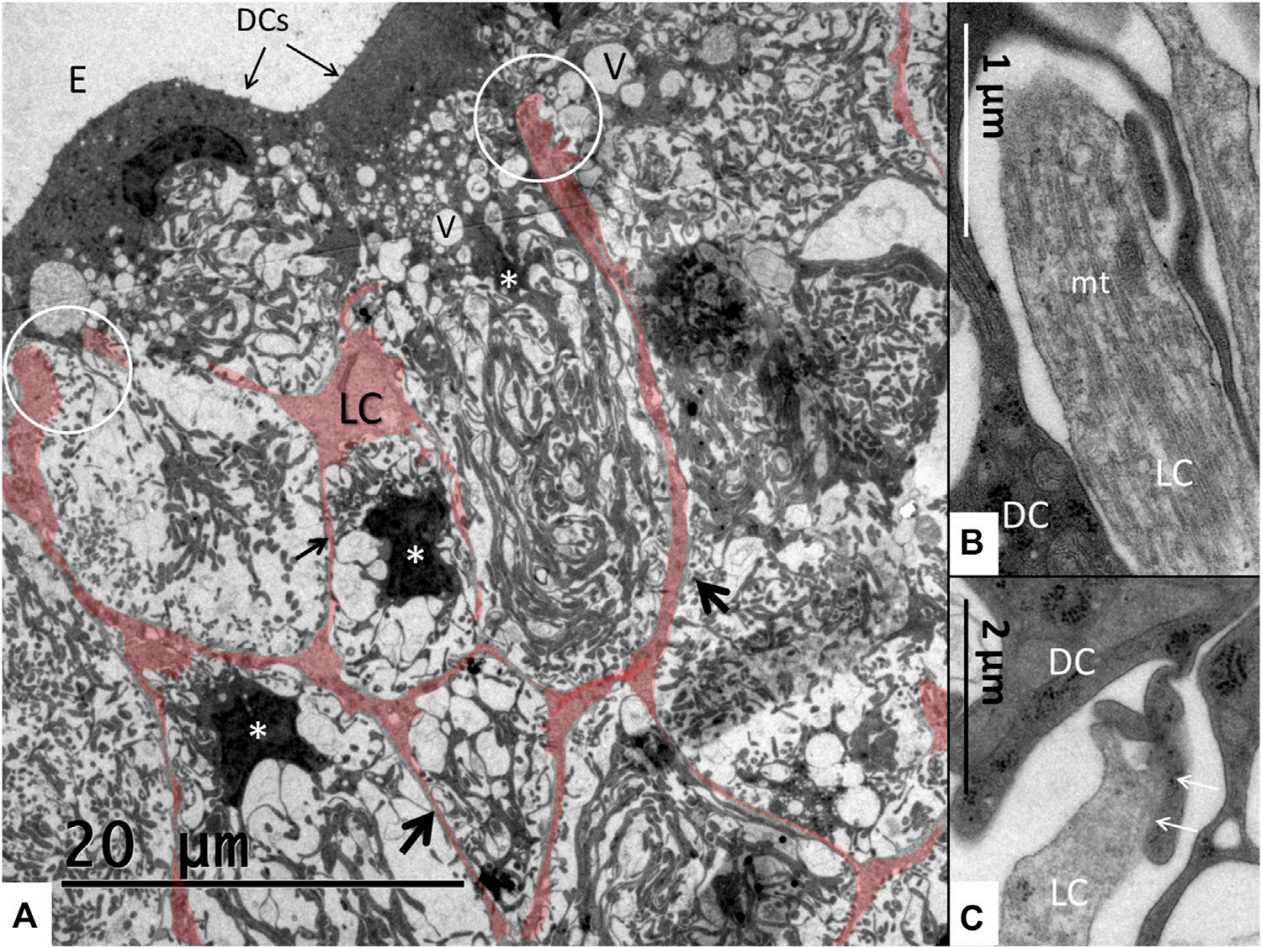
**(A)** Light cells (LCs) are colorized to show the network formation (arrows) forming intercellular spaces containing basal dark cell (DC) enfoldings. Dark cell cytoplasm contains secretory-like vesicles (V) of different sizes. The light cell processes reach apically to the dark cells and form tight junction-like membrane fusions (encircled). Starred cells (*) are epithelial dark cells reaching basally. Their cell nuclei have translocated basally. **(B,C)** Encircled light cell processes are rich in microtubules (mt) and form tight contacts to the dark cells.

**FIGURE 6:**
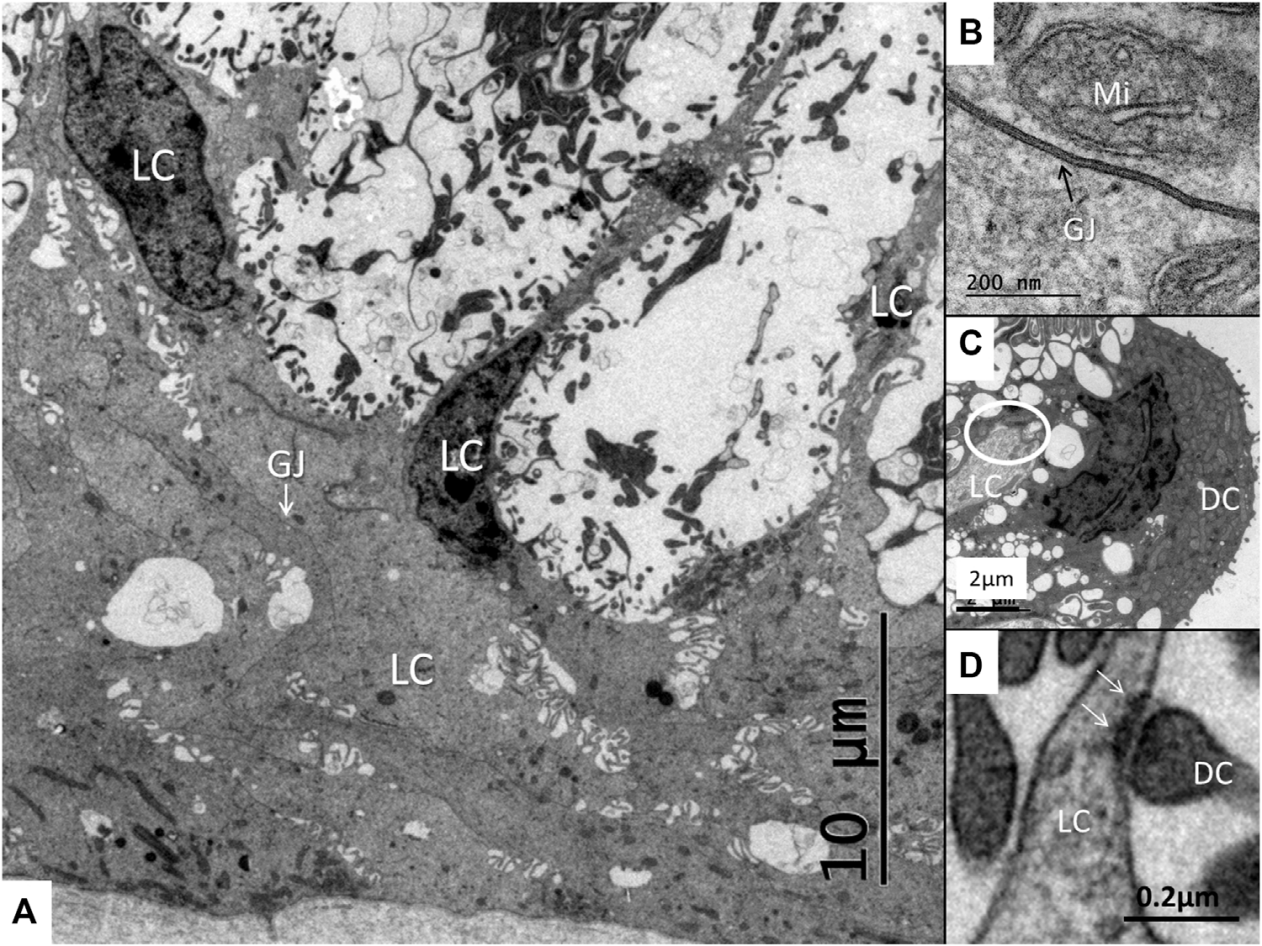
**(A)** Light cells (LC) at the base of the stria vascularis with intercellular spaces containing dark cell enfoldings. The light cells display extensive gap junctions (GJ). **(B)** Higher magnification shows a gap junction between two light cells. Mi, mitochondrion. **(C)** Light cell process (LC) forms an occlusion (encircled) against the dark cell (DC). **(D)** Interaction between the light cell (LC) and dark cell (DC) process within the dilated space. Membrane specialization with focal densities are seen (arrows).

**FIGURE 7:**
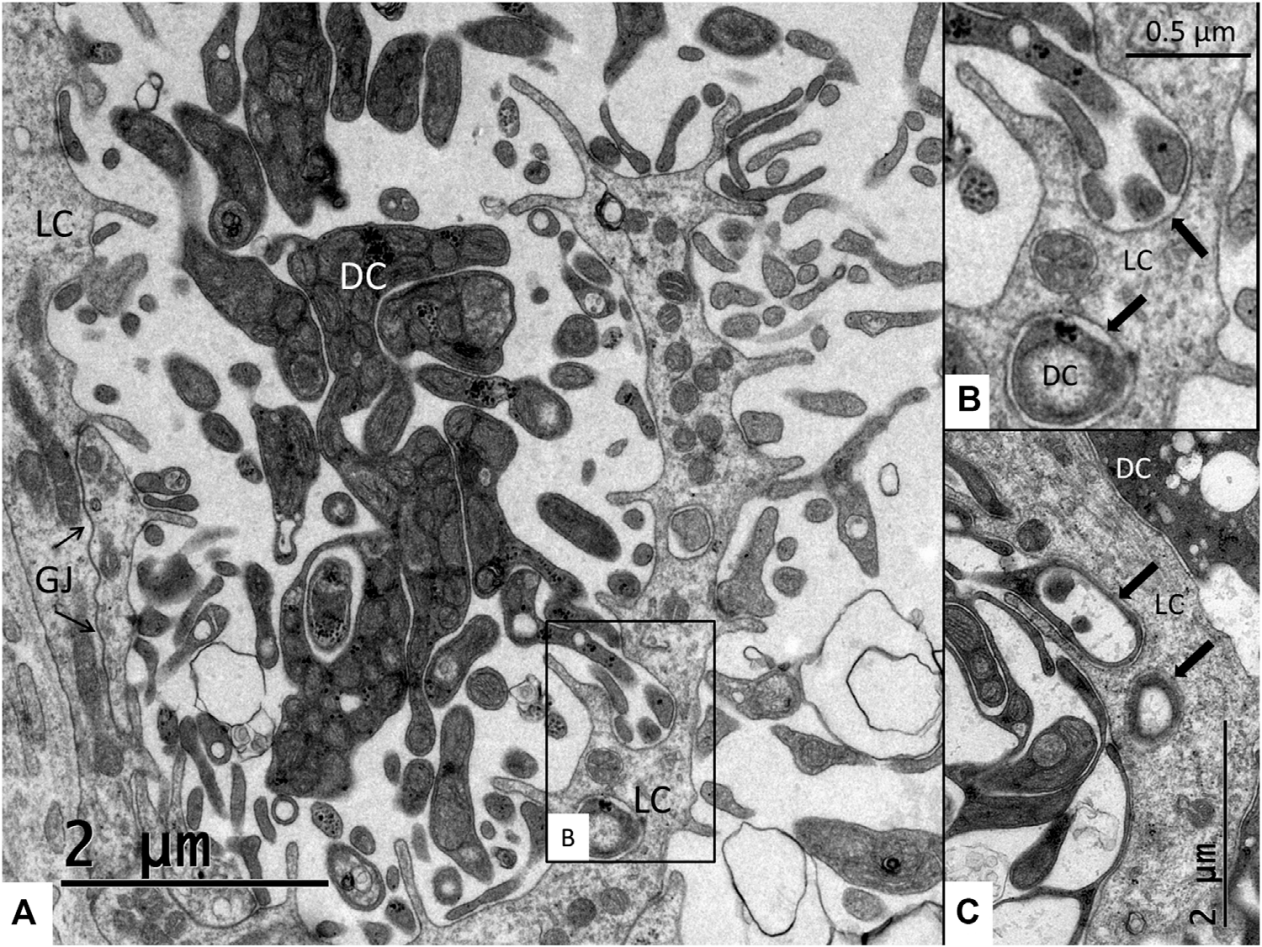
**(A)** Interaction between the light cells (LC) and dark cells (DC). Light cells are connected with extensive gap junctions (GJ). **(B)** Higher magnification of framed area in **(A)**. Dark cell processes project deep into the cytoplasm of the light cells. **(C)** Dark cell processes and light cells membranes adhere close to each other (arrows).

**FIGURE 8:**
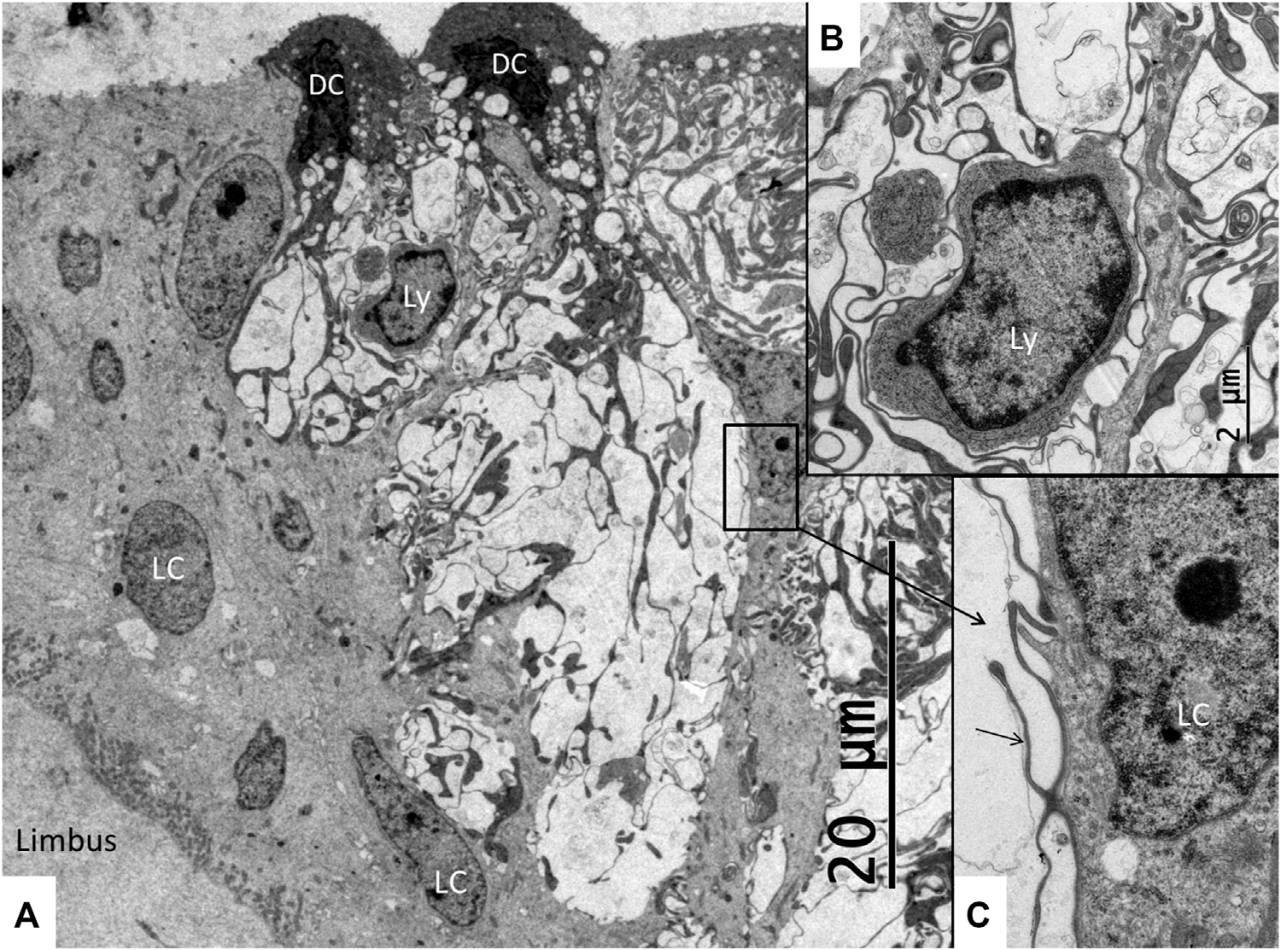
Electron microscopy of the SV near the hyaline cells. Both dark cells (DC) and light cells (LC) are seen forming large intercellular spaces containing dark cell enfoldings. The spaces contain lymphocyte-like cell (Ly) magnified in B and **(C)**. **(B)** Higher magnification of the free cell shown in **(A)**. It is surrounded by dark cell lamellae (arrow) further magnified in **(C)**. **(C)** Higher magnification shows cell lamellae adhering to the cell coat of the lymphocyte-like cell. The light cell nucleus contains a prominent nucleolus.

**FIGURE 9:**
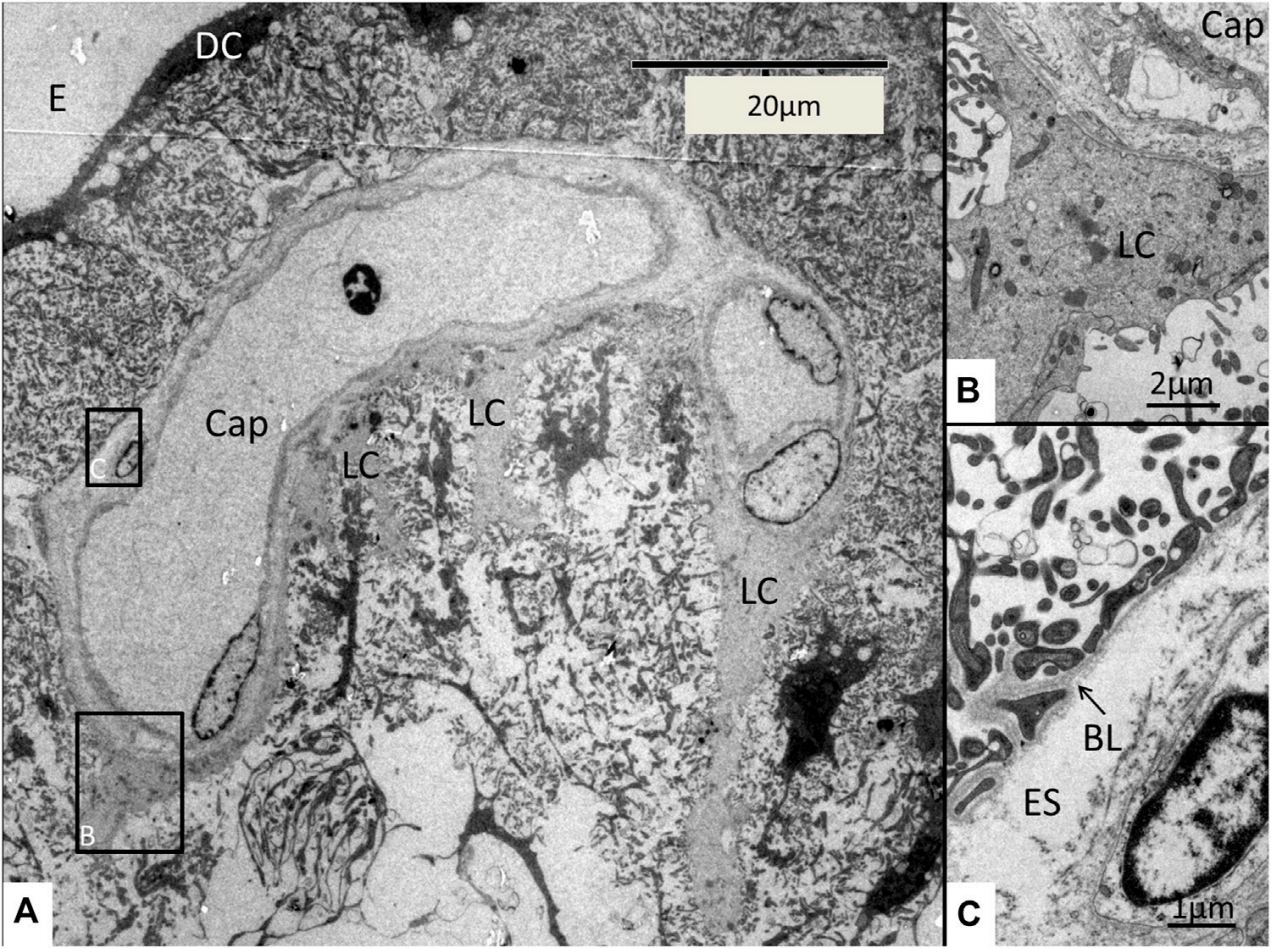
A strial capillary (cap) located near the endolymph space (E). Framed areas are magnified in **(B,C)**. The vessel is partly surrounded by light cells (LC). **(B)** Framed area in **(A)** is magnified and shows a light cell tightly adjoining the capillary wall adhering to the basal lamina. **(C)** Framed area in **(A)** is magnified showing the extensive basal enfoldings of the DCs adhering to the basal lamina (BL) around the pericapillary extra-cellular space (ES). DC, dark cell; E, endolymph.

**FIGURE 10:**
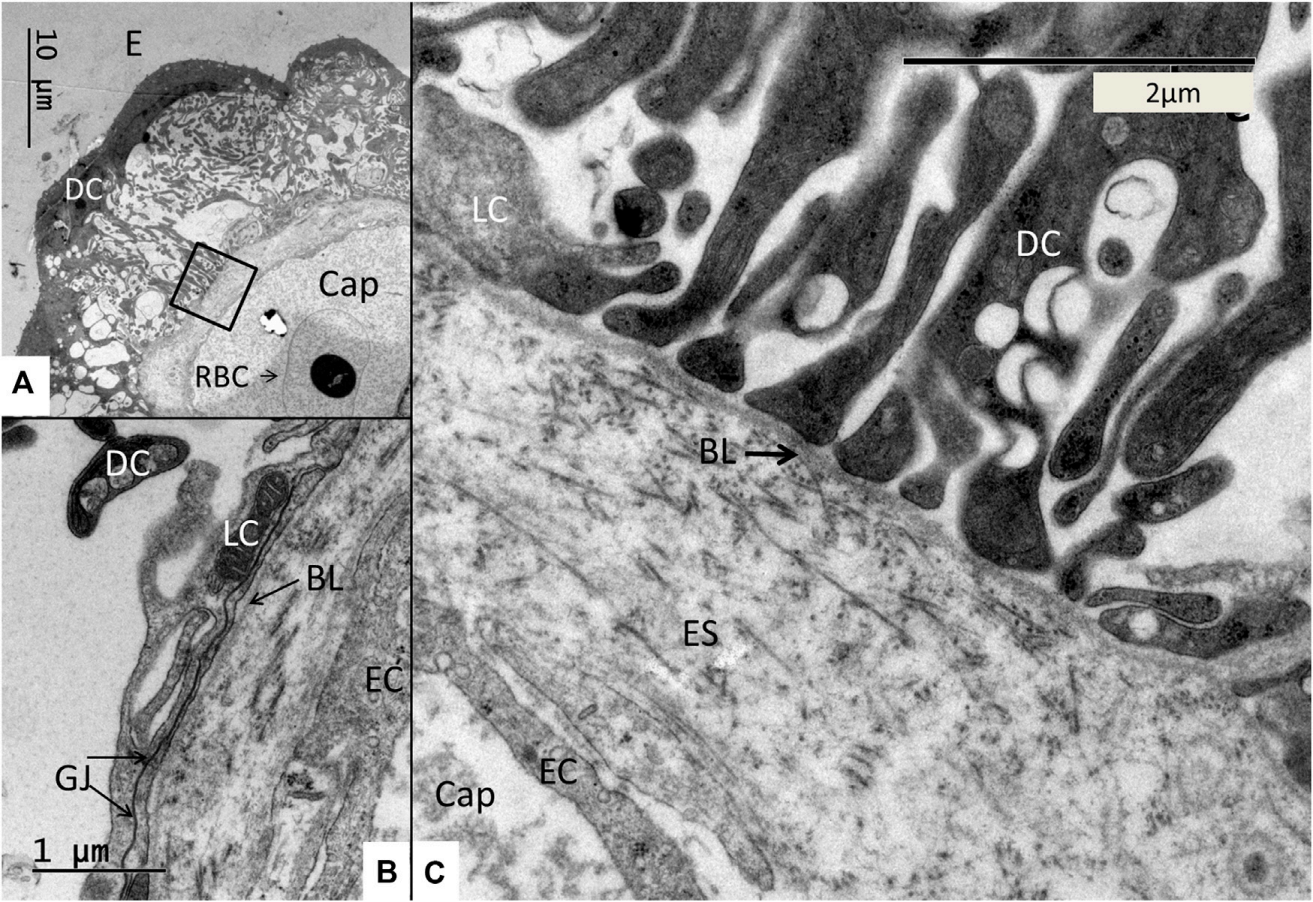
**(A)** Medium-power electron micrograph showing the SV epithelium closely associated with an intra-epithelial capillary. The capillary contains a nucleated red blood cell (RBC). Framed area is magnified in **(C)**. **(B)** Higher magnification shows the pericapillary region with light cells (LC) adhering to a thin basal lamina (BL). The light cells are connected with extensive gap junctions (GJ). There is a space between the endothelial cells (EC) and the basal lamina (BL). This space contains connective tissue fibers. **(C)** Higher magnification shows podocyte-like, dark cells (DC) projections adhering to the basal lamina (BL) and light cells (LC) surrounding the capillary (Cap) endothelium. There is a space containing extra-cellular matrix and thin connective tissue fibers around the endothelium. E; Endolymph. ES; Extra-cellular space.

**FIGURE 11:**
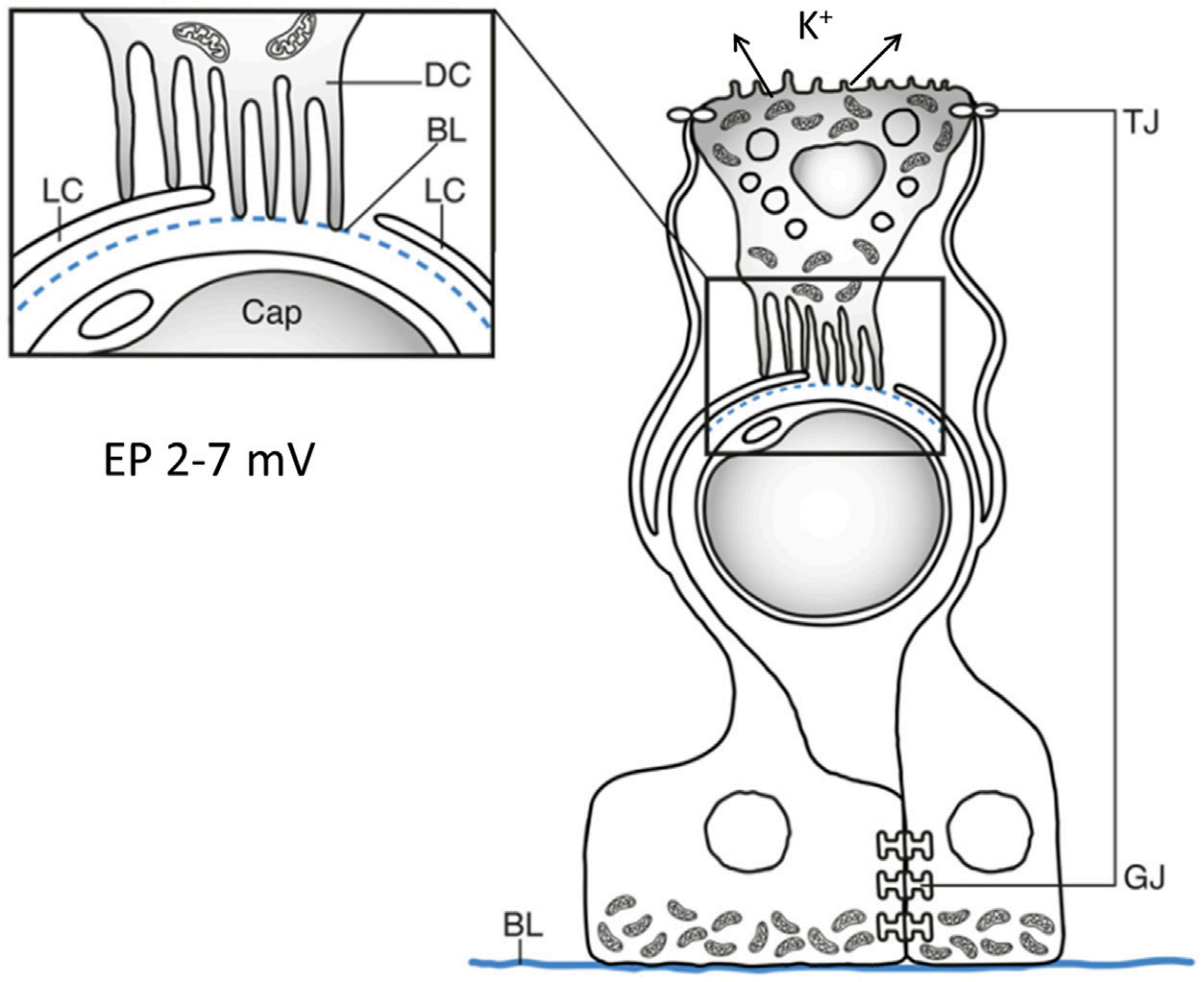
Graphic illustration showing relationship between the SV cells and intra-epithelial capillaries (Cap) and hypothetical representation of endolymph secretion and EP formation in the crocodilian stria vascularis (Crocodylus rhombifer). The dark cell (DC) projections interact with the light cell as well as the pericapillary basal lamina (BL). The light cells (LC) are intercellularly coupled through gap junctions (GJ) allowing ions to be mediated. The light cells form dilated extra-cellular spaces by branches reaching the apical region of the dark cells where they form tight junctions (TJ). These spaces could form a unique environment for the exchange and transport of ions.

The SV epithelium in the *Crocodylus rhombifer* was approximately 75–100 microns in height and consisted of apical electron-dense cells and basal light electron-lucent cells (Figure 3). Laterally, the epithelium extended to the cover fold of the Reissner membrane. The epithelium is located on the vascularized fibrocartilaginous limbus separated by a thin basal lamina through which vessels ran into the epithelium. Electron microscopy showed that the multi-layered epithelium contained several intra-epithelial capillaries. The electron-dense dark cells faced endolymph and showed a multitude of mitochondria almost completely filling the apical cytoplasm (Figure 4A). They also contained clear secretory-like vesicles associated with prominent Golgi structures and rough endoplasmic reticulum. Basally, the dark cells displayed extensive enfoldings or lamellae, less than 40 nm thick, forming complex geometric coils (Figure 4B). The lamellae were dilated only to give space for the large number of mitochondria, sometimes as long as 3 microns (Figure 4C). Their cytoplasm also contained dense ribosomal aggregates. The cellular coils were bathed in large intercellular spaces surrounded by branched light cells forming a network of fluid-filled cavities (Figure 5A). The light cells rested on a thin basal lamina, ramified and extended to the dark cells to which they formed tight junction-like membrane specializations (Figures 5B, C). The light cells were also rich in mitochondria and displayed prominent gap junctions and occasionally round electron-dense bodies (Figures 6A, B). No nerve fibers were detected inside the epithelium except around the hyaline cells as earlier described (Li et al., 2022). The dark cell lamellae often adhered and interacted with the light cells with focal adhesions (Figure 6C, D). Occasionally, lamellae penetrated deep into the light cell cytoplasm (Figures 7A–C). A few round lymphocyte-like cells could be observed in the intercellular spaces. These cells also interacted physically with the dark cell lamellae (Figure 8). The dark basal enfoldings also attached to a basal lamina around the intra-epithelial capillaries (Figure 9) forming palisades of interdigitating podocyte-like processes (Figure 10). The capillaries had a thin endothelium surrounded by a discontinuous basal lamina and an interrupted pericyte sheet. There was a rim of loose extracellular tissue surrounding the vessels containing some fibrous elements margined by a continuous basal lamina (Figures 9B, C, 10). The light cells partly surrounded the capillaries and were interconnected by multiple gap junctions and intimately related to the capillaries (Figures 10B, 11).

**FIGURE 12:**
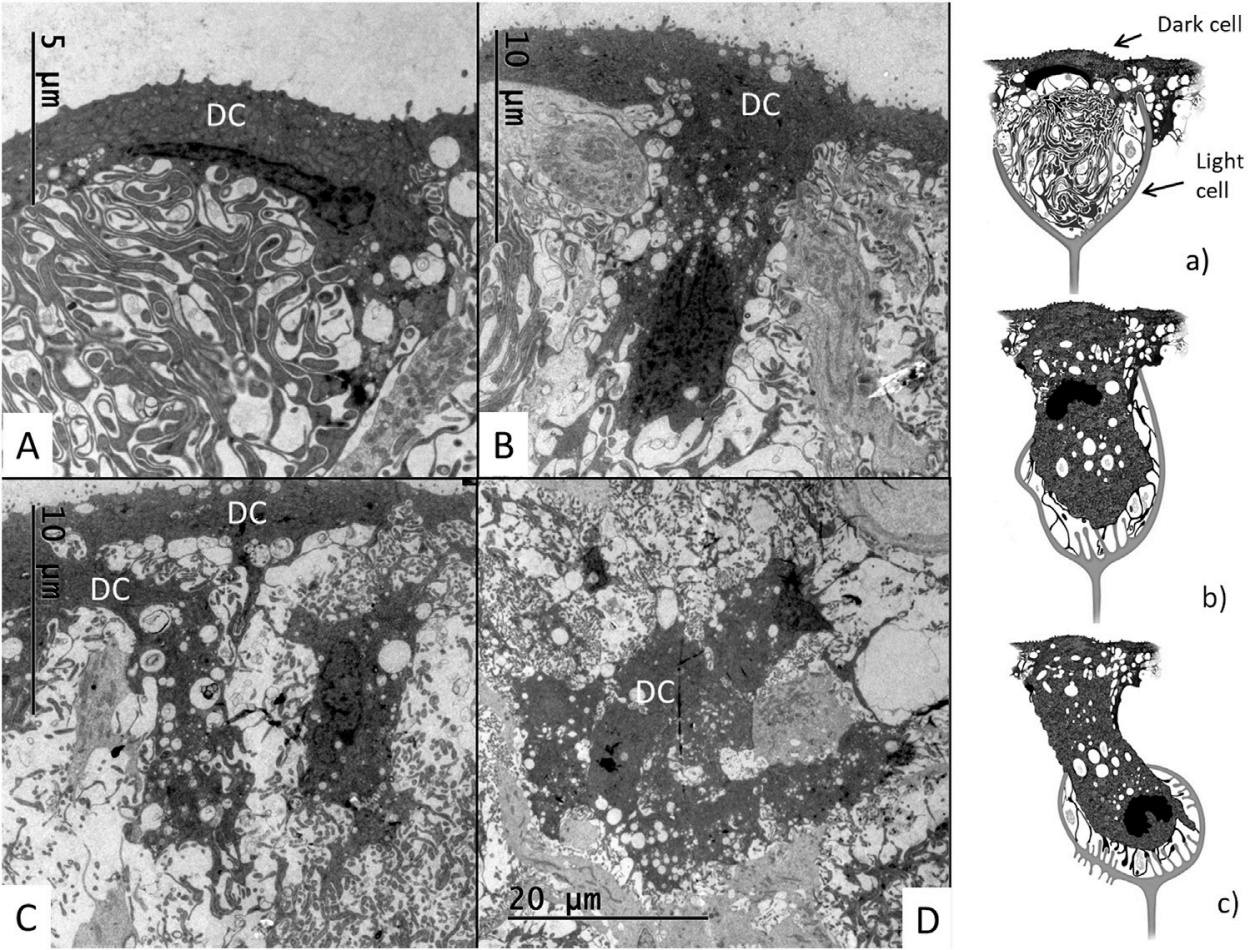
Electron microscopy showing different anatomy of the dark cells in the crocodilian SV. **(A)** A dark cells with thin apical cytoplasm and extensive basal lamellae. **(B)** A T-shaped elongated dark cell projects into the epithelium. Its cell nucleus heads the basal front. **(C)** A dark cell (left) elongates and deviates basally. It contains a large number of clear vesicles and vacuoles assumed to represent secretory vesicles. **(D)** A conglomerate of dark cells are surrounded by light cells. **(a–c)** Illustration of different dark and light cells morphology in the crocodilian SV.

**FIGURE 13:**
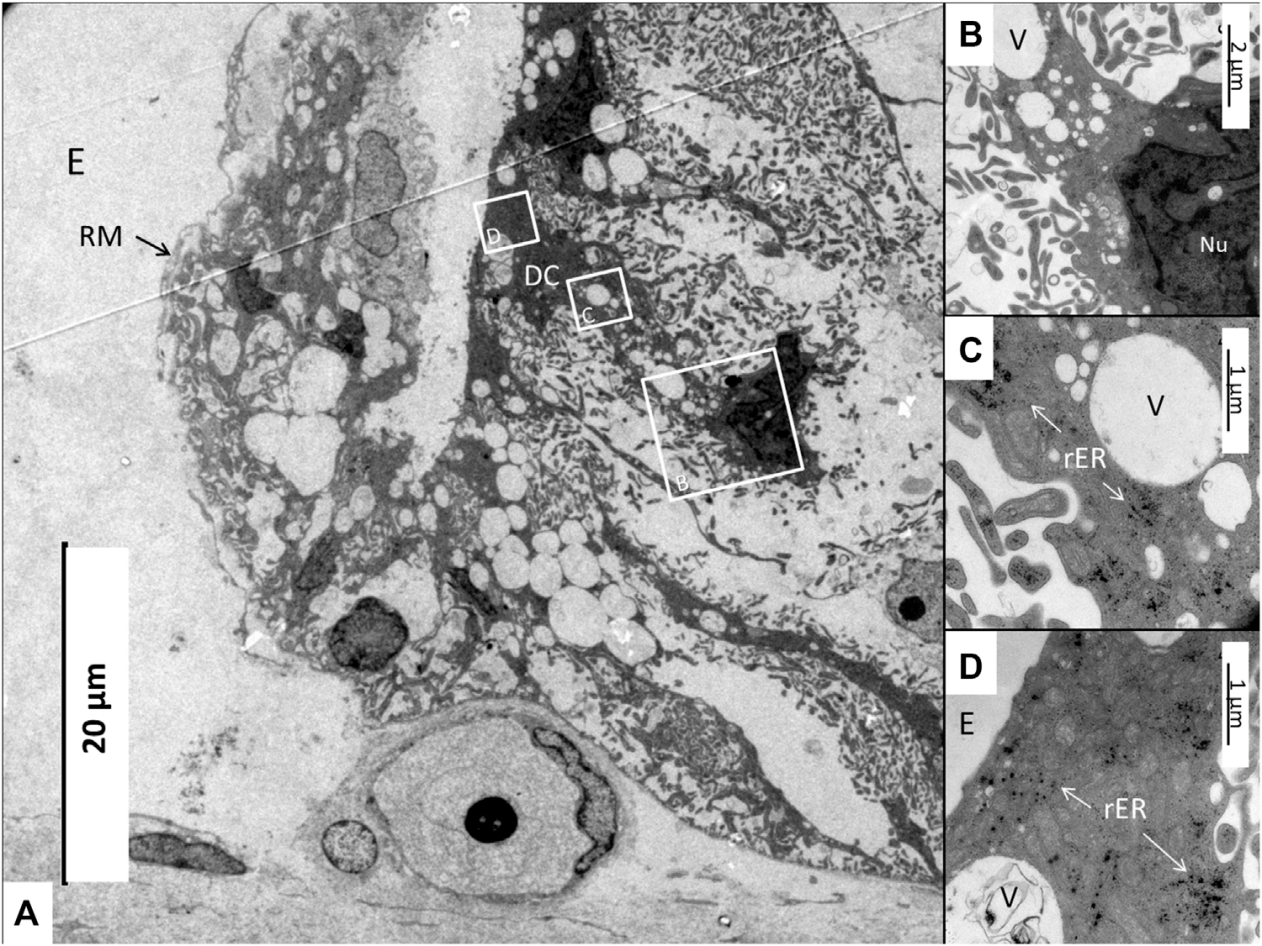
**(A)** Electron microscopy of the stria vascularis near the Reissner membrane (RM) fold. A dark cell (DC, framed) extends basally and its nucleus has translocated. Framed areas are magnified in **(B–D)**. **(B)** Higher magnification of framed area in A shows the cell nucleus (Nu) and a large number of secretory-like vesicles (V). **(C)** Higher magnification of secretory-like vesicles (V) and osmiophilic ribosome aggregates (rER). **(D)** Apical region with multitudes of mitochondria surrounded by ribosome aggregates. E, Endolymph.

The SV contained dark cells also located deeper into the epithelium. Figures 12A–D, a–c show different morphologies of dark cells, sometimes arranged as cell clusters partly surrounded by light cells (Figure 12D). Sections showed that they had expanded basally with the cell nucleus located basally (Figures 12, 13). The dark cells were surrounded by light cell processes forming dilated intercellular spaces at different levels of the epithelium (Figures 12A–C). Serial sections seemed to show that the dark cells maintained connections to the endolymph. The cells had an irregular nuclear envelope (Figure 13B) and often prominent nucleoli. The perinuclear cytoplasm contained many membrane-bound vesicles. Near the endolymph lumen there were large clear, various-sized vacuoles and the cytoplasm showed intensely stained ribosomal conglomerates (Figures 13C, D). Many cells had a close relationship with the capillaries and displayed lateral and basolateral cell processes or lamellae.

### The Reissner membrane and tegmentum vasculosum

**FIGURE 14:**
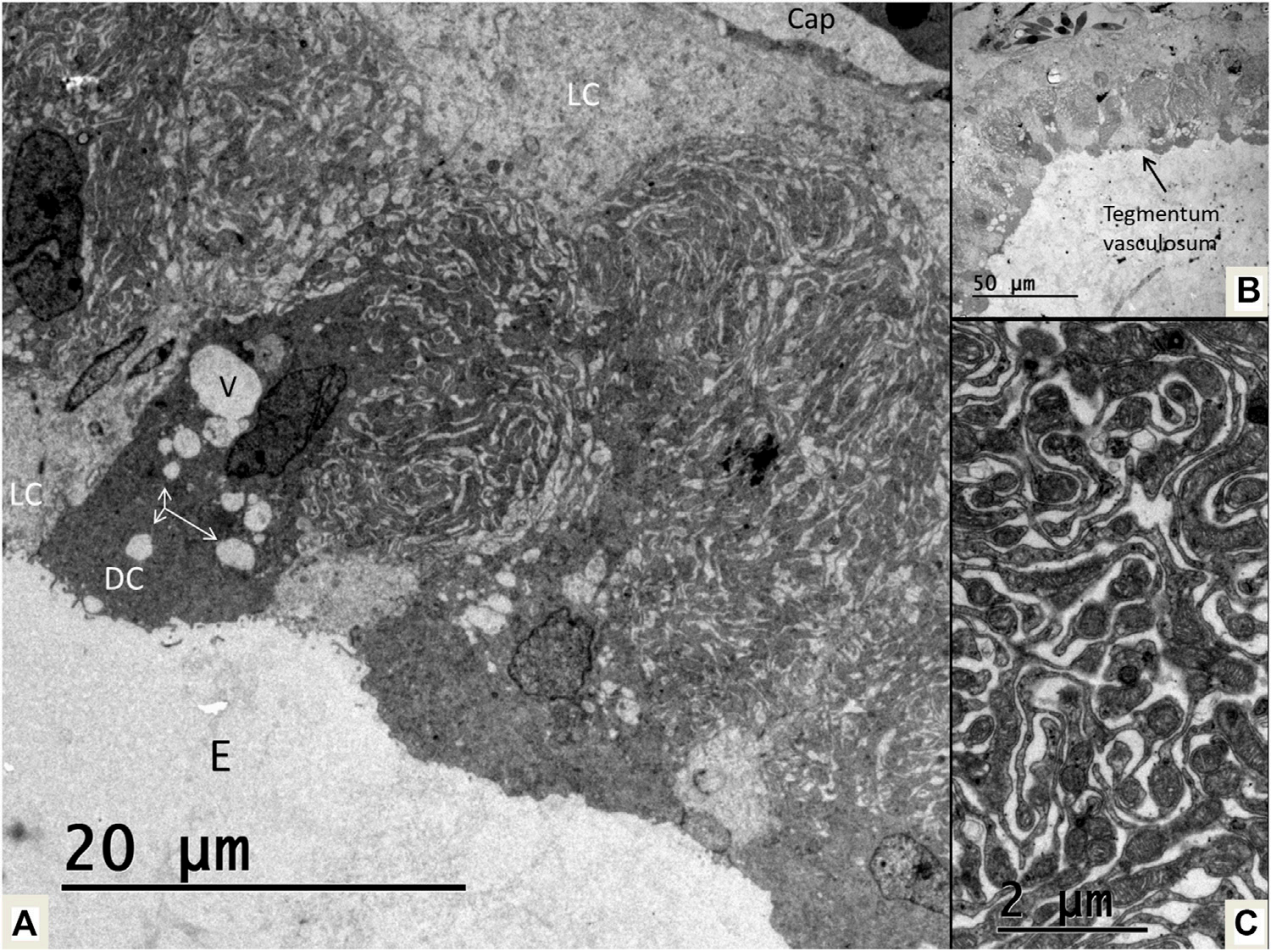
**(A)** Electron microscopy of the tegmentum vasculosum in the crocodile. There are both dark (DC) and light cells (LC). The dark cells contains secretory-like vesicles (V and arrows). Basally light cell face the basal lamina and a subepithelial capillary (Cap). **(B)**. Low power view of the tegmentum vasculosum. **(C)**. Extensive folds of the dark cells contain multiple mitochondria. E, endolymph.

The tegmentum vasculosum also contained dark and light cells (Figure 14). Both cell types reached the lumen. The dark cells had an electron-dense cytoplasm with extensive basal interdigitating enfoldings with many rod-shaped mitochondria of somewhat different appearance than those in the SV. Several blood vessels had subepithelial location. Not invariably, the epithelial cells formed aggregates, and it was not possible to evaluate if the epithelium was single-layered. Basally, it was bordered by light cells forming a rim or cell layer facing the basal lamina. These light cells seemed to be the same as those fronting the lumen and surrounded by dark cells similar to the crocodilian SV. Many dark cells contained clear secretory-like vesicles. The Reissner membrane also contained light and dark cells. Some of these dark cells displayed a large number of thin processes attached to the light cells. Their cytoplasm contained many secretory-like clear vesicles.

## Discussion

In mammals, the SV is an epithelium with unique intraepithelial capillaries containing three functionally different cell types, namely, the marginal, intermediate, and basal cells. Together with the lateral fibrocyte system, they are believed to secrete and recirculate K^+^ ions and generate an EP essential for sensory hair cell transduction (Tasaki and Spyropoulos, 1959; Salt et al., 1987; Wangemann, 2002; Hibino et al., 2010). In a recent investigation, focusing on hair cell regeneration, a separate multi-layered epithelium with intraepithelial capillaries was detected at the lateral fibrocartilaginous plate in the *Crocodylus rhombifer* (Li et al., 2022). Structurally, it is similar to a mammalian SV being vascularized and having both dark and light cells (Smith, 1957; Rodriguez Echandia and Burgos, 1965; Kimura and Schuknecht, 2009). The crocodilian SV showed morphological differences from the mammalian with no joined fibrocyte cell system connected to the epithelium. Melanocyte-like, pigmented intermediate cells were also not clearly recognized. Its location and morphology differed from the TV described in non-mammalian tetrapods such as anurans and birds (Deiters, 1860; Kuhn, 1881; Retzius, 1881; Ishiyama et al., 1970; Hossler et al., 2002b; Mason et al., 2015).

### Earlier studies of the stria vascularis in crocodilians

It was generally believed that crocodiles and birds lack a true SV and that it is substituted by the TV. Retzius (1884) described a similar epithelium consisting of columnar cells at the lateral limbus in the auditory organ of the *Alligator mississippiensis.* An equivalent epithelium was not defined in birds (*Columba livia domestica*). In his detailed illustrations he showed that it contains blood vessels and named it Stria vascularis (Retzius, 1884). In his classical work Das Gehörorgan des Wierbelthiere II, Das Gehörorgan Der Reptilien, Der Vögel und Der Säugethiere” he described this “remarkable” epithelium and stated that he considers this to be the only vascularized epithelium in the body (“Es ist dies Epithel meines Wissens das einzige gefässführende, echte Epithel im Organismus“). Ganeshina (1990) also described a SV located at the abneural limbus in *Caiman crocodilus* (Ganeshina, 1990). She believed it was secretory together with TV and the Reissner membrane suggesting a unique level of morphological differentiation of the secretory apparatus in caimans compared to mammals. All three regions contained dark and light epithelial cells, but the SV epithelium displayed morphological peculiarities with extraordinary intercellular spaces suggesting a separate secretory function. The dark cells were more elongated with extensive luminal surfaces. The giant intercellular spaces were closed by tight junctions. A blood capillary was also mentioned running inside the epithelium near the basal lamina derived from a vessel of the lateral limbus. A few possible nerve fibers were also described in the epithelium. The author concluded that the TV may produce the main volume of endolymph while the SV and Reissner membrane may regulate its delicate ionic composition. Several electron micrographs were presented of the TV but none of the SV.

### The stria vascularis in *Crocodylus rhombifer*

The arrangement of the dark cell lamellae with podocyte-like foot processes adhering to the blood capillaries is similar to that of a nephron glomerulus. Moreover, dark and light cells showed membrane adhesions suggesting absorptive routes. The aqueous spaces could offer optimal conditions for the greatly enlarged surface of the lamellae to absorb fluid and ions to be secreted into endolymph. This machinery could transfer and fine-tune endolymph secretion and EP generation akin to the intermediate and marginal cells of the mammalian SV (Figure 11). The different morphology of the dark cells could suggest that they are highly dynamic cells possibly even “amoeboid-like” able to expand deeper into the epithelium still maintaining exchange to the endolymph surface. It could increase the potential to process and secrete endolymph fluid.

The light cells resemble intermediate perivascular cells that are essential for the generation of high EP in the mammalian SV (Hibino et al., 1997; Wangemann, 2002; Hibino and Kurachi, 2006; Liu et al., 2017). Intermediate cells are known to express the inward rectifying potassium channel Kir4.1. Gap junctions were prominent between the light cells suggesting exchange of ions, small metabolites, and second messengers between adjacent cells (Figure 11). They may facilitate K^+^ transport to the dark cells and could suggest that the light cells have a function also similar to the basal cells in the mammalian SV. The light cells in the crocodile could therefore be analogous to both the basal and intermediate cells to relay K^+^ ions for endolymph secretion and generation of EP. The intercellular spaces formed by the light cells in the crocodile could be analogous to the electrically isolated intrastrial space in mammals that are surrounded by marginal and intermediate/basal cells indispensable for maintaining EP (Nin et al., 2008). Further examinations are required to establish the involved ion transporters as well as the molecular composition of the gap junctions in the crocodilians.

### The tegmentum vasculosum

The *Crocodylus rhombifer* displayed a prominent but variable TV that overlay the papilla basilaris as earlier demonstrated in the *Alligator mississippienses* and *Caiman crocodilus* (Retzius, 1884; Ganeshina, 1990). Retzius’ illustrations show a tegmentum of varying size along the papilla basilaris, but it seems to be missing in some regions. Deiters (1860) and others described this peculiar epithelium and indicated its analogy to the mammalian SV (Deiters, 1860). It was folded and highly vascularized with subepithelial vessels in anurans, neobatrachians, and birds (Hasse, 1873; Kuhn, 1881; Retzius, 1881; Retzius, 1884; Satoh, 1917; Held, 1926; Dohlman, 1964; Jahnke et al., 1969; Dohlman, 1971; Smith and Takasaka, 1971; Mason et al., 2015; Wilms et al., 2016). It is believed to secrete endolymph and generate EP, and its spectacular ultrastructure was exquisitely demonstrated in birds (Ishiyama et al., 1970; Rosenhall, 1971; Hossler et al., 2002a; Hossler et al., 2002b). Extensive basolateral dark cell enfoldings were described with many mitochondria similar to the crocodilian SV. Not only similarities but also differences between the TV, vestibular system, and marginal cells of the mammalian SV were highlighted. Typically, a basal lamina separated light and dark cells from the connective tissue and blood vessels (Ishiyama et al., 1970; Hossler et al., 2002b). Na/K-ATPase activity was demonstrated in the chicken TV similar to the dark cells of mammalian SV (Kuijpers et al., 1970; Yoshihara et al., 1987). Wilms et al. (2016) also identified several deafness genes encoding ion channels and transporters known to be expressed in the mammalian SV. They concluded that both the TV and SV may have evolved from an ancestral vestibular type of epithelium. GJB2 and GJB6, which are important human deafness genes encoding connexin26 and connexin30, were also expressed. The gene encoding the inward-rectifying potassium channel Kir4.1, known to be essential for the formation of high EP in mammalian SV, could not be localized in the avian TV (Tanaka and Smith, 1978; Hibino et al., 2010; Wilms et al., 2016). In the present investigation, the Reissner membrane epithelium also contained dark cells with digitating processes and secretory-like clear vesicles. Therefore, all secretory-like epithelia in the crocodilian seem to share some similar morphological signatures.

### Crocodiles and the evolution of the endocochlear potential

The mammalian cochlea has an exceptionally high EP playing an important evolutionary role (Manley, 2012; Köppl and Manley (2019)). It is believed to be generated in the multi-cellular compartments of the SV by an electrogenic component generated by active ion transport across the perilymph-endolymph barrier and K^+^ diffusion potentials (Kuijpers et al., 1970; Salt et al., 1987; Wangemann, 1995; Sauer et al., 1999; Nin et al., 2008; Hibino et al., 2010). It is developed during the period of cochlear elongation and high-frequency hearing. Studies in the pigeon and crocodiles, including caiman crocodilus (*Paleosuchus trigonatus*, *Melanosuchus niger*, *Caiman crocodilus*), show that cochlear microphonics and large summating potentials can be detected even in the absence of a large DC potential (Schmidt and Fernandez, 1962). Low hearing thresholds and increased sensitivity were recorded even in animals with low EP. Moreover, an EP may not entirely depend on a developed SV. Some birds, especially the barn owl, have a high anoxia-sensitive EP and possess a large TV (Wilms et al., 2016; Köppl, 2022). Crocodilians are ectothermic animals and anoxia-insensitive with a low EP (Schmidt and Fernandez, 1962) that could be linked to their low metabolic rate, respiratory differences, and adaption to different aquatic environments (Wilms et al., 2016; Köppl et al., 2018). The EP constitutes the driving force for K^+^ across the transduction channels, and, similar to mammals, caiman crocodilus have an inner ear travelling wave, and there is also active mechanical processes within the basilar papilla (Wilson et al., 1985). *Crocodylus rhombifer* surprisingly expressed the anion transporter prestin in both the long and short hair cells suggesting a voltage-dependent electromotility or active amplification driven by voltages across the hair cell membrane (Li et al., 2022). Recordings of stimulated otoacoustic emissions showed a non-linear growth with stimulus intensity and frequency-selective suppression characteristics in the caimans (Wilson et al., 1985). It may improve sensitivity to higher frequencies even though characteristic frequencies of single auditory neurons in the caimans seems restricted from 70 to 2,900 Hz with tonotopic organization of central neurons similar to birds (Manley, 1970; Wilms et al., 2016). Furthermore, the tight cellular organization within the crocodilian basilar papilla may restrict active hair cell motion and alternate system of hair bundle gating could play a more prominent role.

The size of the SV seems to vary among the investigated crocodile species, possibly explained by the different regions that were analyzed (Retzius, 1884; Baird, 1974; Ganeshina, 1990). SV in caimans was restricted to the proximal part of the cochlear duct (Ganeshina, 1990) that may suggest that gradients exist along the papilla to monitor endolymph microchemistry. Variations at different frequency locations may reflect the existing gradients in endolymph composition, receptor currents, and EP, serving to optimize hearing sensitivity at different hair cell regions as suggested by Ganeshina (1990). Gradients in Na/K-ATPase activity were shown in the mammalian cochlea being larger in the basal turn compared to the apex (Kuijpers et al., 1970; Salt et al., 1987; Sziklai et al., 1992).

Crocodiles and birds belong to the archosaurs dating back to the dinosaurian era (Brusatte et al., 2015). Their well-developed hearing and communication skills imply intriguing steps in the evolution of the amniotic ear. Crocodiles and birds have anatomically similar inner ears but only crocodilians seem to have developed a SV (Takasaka and Smith, 1971; Baird, 1974; Oesterle et al., 1992; Köppl, 2022). This is surprising, considering its assumed function is to generate an EP, which is larger in birds than in crocodilians (Schmidt and Fernandez, 1962). It may suggest that the TV, which is well developed in both birds and crocodilians, also plays an important role for the generation of EP. Crocodilians depend on the external physical environment to a high degree, while birds have an endothermic (“warm-blooded”) metabolism. Birds are believed to have expanded greatly after the mass annihilation about 66 million years ago (Brusatte et al., 2015). Genetic evolution suggests that nearly all modern birds developed during a relatively short period thereafter (Jarvis et al., 2014). Dinosaurs are believed to be intermediate (“mesothermic”) (Padian and Horner, 2004) and may have been more like large living birds and mammals than reptiles. Consequently, crocodilians could have adopted particular instruments to adapt to more variable aqueous-terrestrial habitats, such as under water and in low temperature conditions, which have led to modifications of their inner ear secretory epithelia.

Hearing depends on a strict homeostatic regulation of the ionic and micro-chemical environment around the hair cells. In mammals, a blood-labyrinth and tight junction barrier exists (Claudin-11) that protects the inner ear to some degree from fluctuations in homeostasis (Gow et al., 2004; Kitajiri et al., 2004; Liu et al., 2017). In the crocodile, endolymphatic membranes, such as the Reissner membrane and TV, are well vascularized, seemingly lacking such barriers, making them theoretically more vulnerable to external conditions. However, a tight junction barrier looks to exist between the light and dark cells to create an isolated environment in the dilated spaces. This may correspond to the unique intrastrial compartment of the mammalian SV essential for the generation of the electric field potential. Crocodilian species possess extracellular osmoregulation through specific salt excreting glands (Taplin and Grigg, 1981). *Alligator mississippiensis* live in freshwater ecosystems and lack salt-secreting glands that are present in Crocodylidae. Different marine/estuarine habitats, ecological niches, and isolated cave ecosystems (Shirley et al., 2017) could increase requirements for supplementary systems to fine-regulate endolymph homeostasis and optimize hearing. This may explain the different anatomy and evolution of the inner ear secretory epithelia in reptiles and birds. The crocodilian SV seems to be an ancient functional innovation and may constitute example of an intriguing parallel evolution through different selective pressures.

## Conclusion

Transmission electron microscopy shows that the auditory organ in *Crocodylus rhombifer* contains a SV separate from the TV. Here, we show the cellular organization for the first time. The SV is believed to secrete endolymph and participate in the generation of the EP, essential to increase crocodile hearing sensitivity under various environmental conditions. The crocodilian SV may constitute example of an intriguing parallel evolution through different selective pressures alongside the TV.

## Data Availability

The original contributions presented in the study are included in the article/supplementary material, further inquiries can be directed to the corresponding author.
